# Spectroscopic approaches for structural analysis of extracted chitosan generated from chitin deacetylated for escalated periods

**DOI:** 10.1186/s13065-025-01558-3

**Published:** 2025-07-16

**Authors:** S. N. Ghanem, M. I. Marzouk, M. E. Tawfik, S. B. Eskander

**Affiliations:** 1 Department of Polymers and Pigments, National Research Centre, Cairo, Egypt; 2 Faculty of Science, Chemistry Department, Ain Shams University, Cairo, Egypt; 3 Radioisotopes Department, Egyptian Atomic Energy Authority, Cairo, Egypt

**Keywords:** Shrimp shell waste, Chemical extraction of Chitosan, Degree of deacetylation, FTIR-ATR, SEM-EDX, UV-Vis., TG, DTG, DSC

## Abstract

A conventional chemical method was applied for the extraction of chitosan (CH) from shrimp shell wastes (SSWs) in three stages: (1) Demineralization: SSWs were treated with HCl to remove minerals. (2) Deproteinization: NaOH was used to eliminate proteins from the demineralization shells. 3: Deacetylation: The chitin (CT) obtained from stage 2 was converted to chitosan in alkaline medium using NaOH. This study aims to demonstrate the impact of varying deacetylation times on chitosan surface morphology, elemental composition, thermal resistance, structural configuration, and deacetylation degree (DD). Variable techniques including UV-visible spectroscopy, Fourier Transformed Infra-Red (FTIR-ATR), Thermogravimetry (TG/DTG), Scanning Electron Microscopy (SEM), and Energy-dispersive X-ray spectroscopy (EDX) analyses were employed to analyze how increased deacetylation periods affect the characterization of the products. The FTIR spectra showed a notable similarity between all extracted chitosan processed with increasing deacetylation time and the commercial one. Moreover, the results revealed that all the extracted chitosan samples acquired DD values, based on FTIR-ATR analysis, are comparable to that of commercial ones i.e. 79.54%, 78.23%, 74.81%, and 76.56% for deacetylated times of 22 h, 30 h, 36 h, and 40 h, respectively are comparable to that of the commercial chitosan (76.1%). Furthermore, the EDX analysis confirms that the extracted chitosan is non-toxic product, making it suitable for various applications, including biological and medical uses.

**Graphical Abstract:**

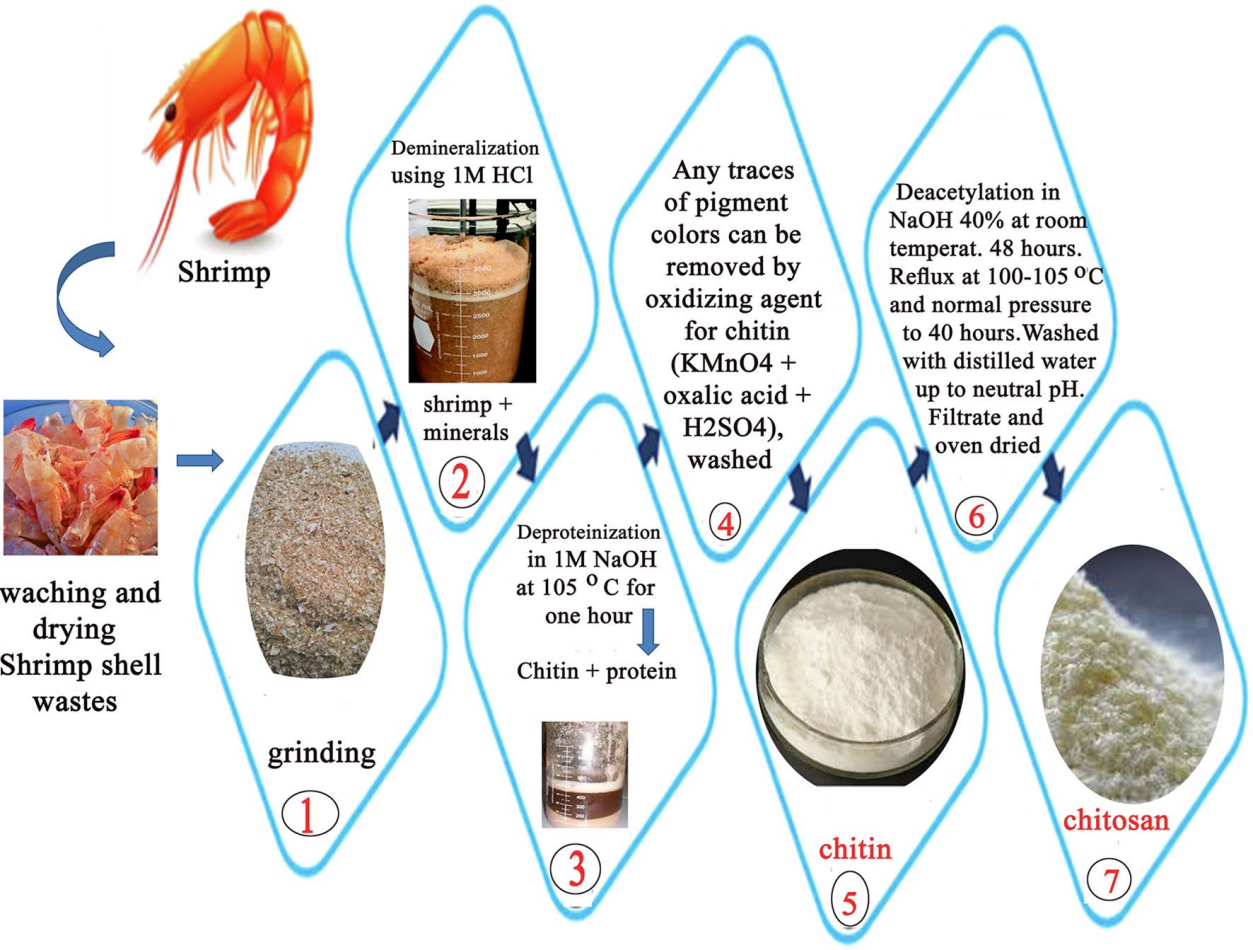

## Introduction

The name chitosan designates a chain of deacetylated chitin macromolecules with diverse molecular weight, viscosity, and degree of deacetylation, (DD) (40–99%). Chitosan is a linear polyamine with a number of amino groups that are accessible for chemical reactions with acids or other active groups [1]. It is a cationic amino polysaccharide and it has extensive applications in many fields: in medicine, biomedical and pharmaceutical industries e.g. fibers, membranes, and artificial organs; in biology; in agriculture; in the environment; in the field of cosmetic production alike body creams, lotions, additives for the hair; in addition to some foodstuff manufactures e.g. binder, gelling agent, thickener; antimicrobial agents and antioxidants fabrication are among others [2, 3]. Chitosan has great attention among researchers, due to its efficient scavenger capability for heavy metals [4–7]. Chitosan beads can be applied, also, to sorb radionuclides e.g. radiocesium, radiocobalt…etc., from radioactive aqueous solution streams [8, 9].

Different practices have been applied to extract chitin and consequently chitosan, but the furthermost widespread is the chemical processing route [10].

Many analysing tools are used to characterize the chitosan products, among all those methods: Fourier Transformed Infra-Red, Thermal analyses, and Scanning Electron Microscope prove to be a very beneficial tool since it was applied to reveal the amount and the nature of chemical fractions generated from various composite materials in addition to DD evaluation [11–13].

In this study all-chitosan products extracted from shrimp shell wastes are subjected to characterization using UV-visible spectroscopy, Fourier Transformed Infra-Red (FTIR-ATR) examination, Thermogravimetry, Derivative thermogravimetry (TG/DTG), Differential scanning calorimetric analyses (DSC), Scanning Electron Microscopy (SEM), and Energy-dispersive X-ray spectroscopy (EDX) analysis. Those tools were applied to evaluate the impact of increasing deacetylation periods on the physico-chemical properties of the obtained chitosan and to compare it to the commercial chitosan purchased from Sigma Reagent Co.

## Materials and methods

### Materials

Fresh samples of shrimp shells were obtained from the wastes of processing discards and collected from a local shrimp market in Egypt. The shrimp shell wastes (SSWs) under consideration, were selected, packed in plastic bags, and stored at (-10 °C) before and during transportation to the laboratory. The SSWs were washed with water and soap several times, boiled with water and soap for 10 min, rewashed, and then dried in an oven at 90 °C. Finally, the clean dried shells were ground to the predetermined smallest mesh sizes.

Commercial chitosan with 76% deacetylation degree was purchased from Sigma Reagent Co., Ltd. It was used as a reference for comparison with the extracted one. All other used chemicals in this study were of analytical grade and applied without any further purification.

## Methods

### Extraction of chitosan

#### Demineralization step (1)

The demineralization of ground shrimp shells was carried out at room temperature (25 ± 5 °C) using 1 M hydrochloric acid until the production of carbon dioxide (CO_2_) gas was completely stopped. The demineralized ground shells were filtered under suction through a Buchner funnel with coarse porosity filter paper Whatman No. 40, and the residue was washed with distilled water several times till it reached neutral pH (6.5 ± 0.5). Finally, the solid material was dried in a drying oven at 90 ºC for constant mass. The chemical reaction for this step can be illustrated as follows: the minerals in the shells (mainly calcium carbonate) are decomposed into calcium chloride along with the discharge of carbon dioxide, by hydrochloric acid at room temperature.

1 $$2 \mathrm{HCl}+\mathrm{CaCO}_3 \rightarrow \mathrm{CaCl}_2+\mathrm{H}_2 \mathrm{O}+\mathrm{CO}_2 \uparrow$$

#### Deproteinization step (2)

Deproteinization of the demineralized SSWs was carried out using 1.0 M sodium hydroxide solution (NaOH) (1:10 mass/v ratio of demineralized SSWs to NaOH) at (100 ± 5) °C for one hour. This step was repeated several times. The absence of protein was indicated by the absence of a pale pink color in the medium. The product was rinsed with distilled water up to neutral pH. At this stage, perfectly white chitin is isolated. Any traces of pigment colors can be removed by applying a mild oxidizing agent for chitin (KMnO_4_ + oxalic acid + H_2_SO_4_), washed with distilled water, and then dried in an oven at 90 °C [9].

#### Deacetylation of chitin step (3)

The deacetylation of chitin can be highly facilitated by steeping it for 48 h in concentrated sodium hydroxide solution, (40% by mass) at room temperature (25 ± 5) °C before heating. Then, the container used for deacetylation was subjected to reflux at (100 ± 5) °C, at normal pressure, and up to 40 h; the reaction was accompanied by drastic degradation of the chitin-producing chitosan. The obtained chitosan was washed with distilled water to a neutral pH and oven-dried at 90 °C for constant mass [9, 10].

#### Evaluation and analyses

##### Fourier-Transform infrared spectroscopy (FTIR-ATR)

The most interested functional groups of the prepared samples were determined by Fourier-Transform Infrared Spectroscopy with Attenuated Total Reflectance (FTIR-ATR), Bruker Vertex 80 V with resolution 4 cm^-1^ in the range of 4000–400 cm^-1^. The sample was examined directly in a powder form without preparation.

##### Scanning Electron microscope and Energy-dispersive X-ray spectroscopy (SEM-EDX)

The surface morphology of shrimp shell, chitin, commercial chitosan, and extracted chitosan acquired from chitin deacetylation for increasing periods were investigated by applying the surfaces of small pieces to field-emission scanning electron microscope (FE-SEM, Quanta FEG-250) at 20Kv after covering with a thin layer of gold. The samples were subjected to the dry tiny pieces of the various samples to gold plating in S150 a Sputter Coater Edwards (England).

Predetermined samples were subjected to analysis by Energy-dispersive X-ray spectroscopy (EDAX) AMETEK Materials Analysis Division.

##### Ultraviolet / Visible spectroscopy

UV/visible absorption spectra (200 to 900 nm) were recorded by Cary-100 UV-2450 spectrophotometer using a slit width of 2 nm. A pair of quartz cuvettes with a path length of 1 cm was employed for this purpose. Samples of commercial chitosan and extracted chitosan acquired from chitin deacetylation for increasing periods were dissolved in 0.1% acetic acid solution and absorbance spectra of the samples were measured at above selected wavelength ranges. Duplicate measurements were made for each sample and the average values were reported.

##### Thermal analysis

Thermogravimetric (TGA) Differential Thermogravimetric (DTG) and Differential scanning calorimetric analyses (DSC) were performed using Setaram KECHNOLOGIES Themys one (France). Each analysis was performed in a platinum cell under a nitrogen atmosphere at a flow rate of 20 ml/min starting from room temperature up to 600 ^o^C at a heating rate of 10 ^o^C /min.

## Results and discussion

The chitosan can successfully be prepared from the deacetylation of chitin which is extracted chemically from shrimp shells. The quality and physicochemical properties of prepared chitosan are varied widely with the quality of shrimp shell, chitin, and methods of preparation. The production of chitosan is based on the deacetylation reaction of chitin i.e. of hydrolysis of chitins’ acetamide groups. This is associated, also, with the splitting of chitin’s acetyl group with the formation of an amino group. Additionally, the deacetylation reaction can be associated with a concurrent rupture of the polymer glycosidic bonds.

### FTIR -ATR analysis

**Fig. 1:**
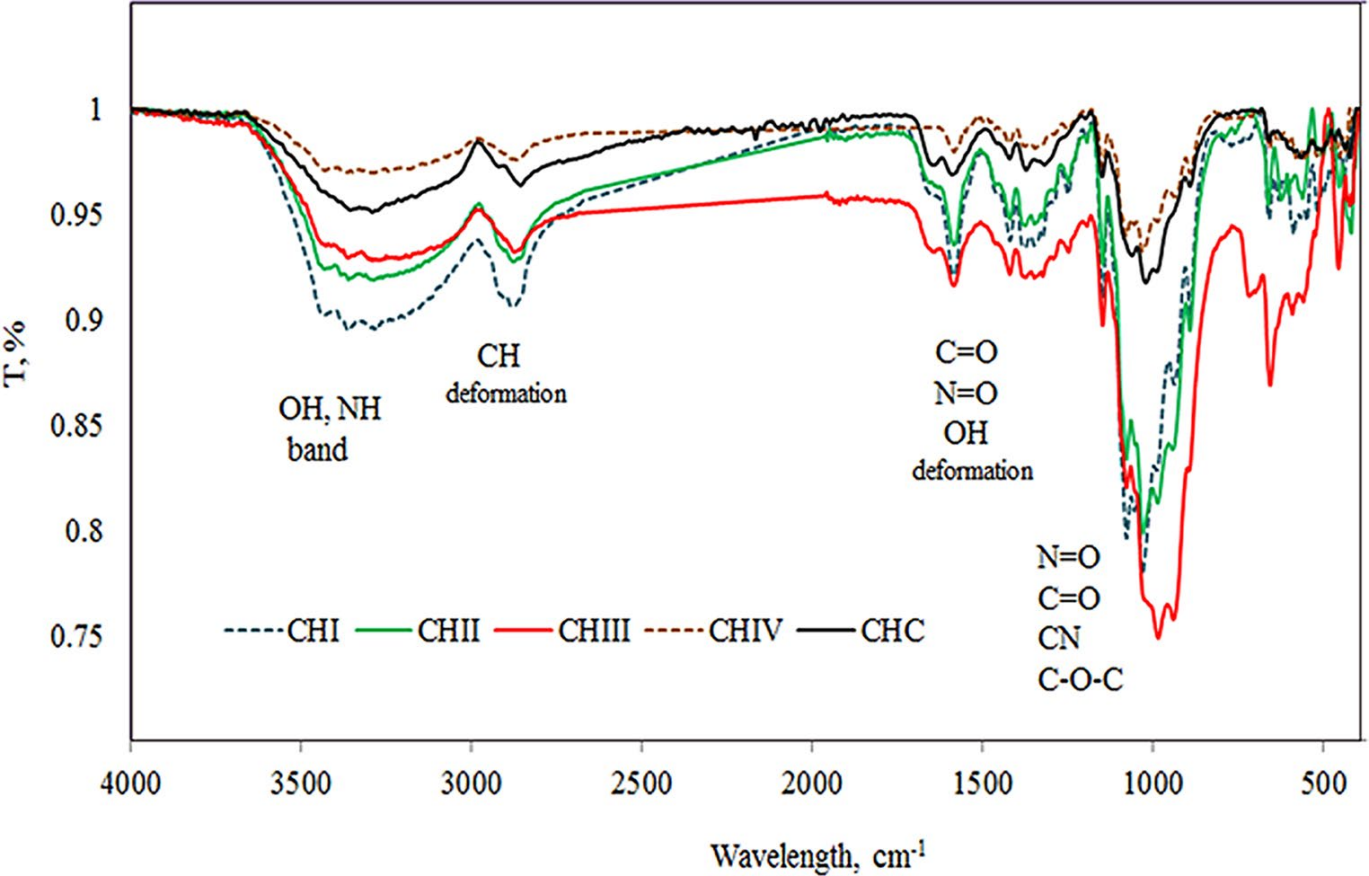
FTIR-ATR spectra of commercial and various extracted chitosan acquired from chitin subsequent to increasing deacetylation periods time

Infrared spectroscopy is one of the highly interesting and extensively applied analytical practices, based on the vibrations of the atoms of a molecule, to study the chemical configuration of materials. Moreover, FT-IR analysis can be used to evaluate the degree of deacetylation of chitosan extracted from shrimp shell waste. In this study, FTIR-ATR analyses were performed systematically for commercial chitosan (CHC) for comparison, besides for the extracted chitosan acquired from chitin following increasing deacetylation periods i.e.: 22 h (CHI); 30 h (CHII); 36 h (CHIII), and 40 h (CHIV) under the same analyses’ conditions, to evaluate the impact of escalation of deacetylation periods on DD % and their spectra. The results obtained are presented in Figure. 1 and Table 1.

**Table 1:** The acetylation (DA %), deacetylation (DD %) percentages and molecular weight (MWT) of commercial and various extracted chitosans acquired from subsequent chitin deacetylation for increasing periods

Deacetylation time, hour	22	30	36	40	CHC***
DA*, %	20.46	21.77	25.19	23.44	23.89
DD**, %	79.54	78.23	74.81	76.56	76.11
MWT****, g/mol	15,280	14,957	13,349	14,421	41,300

*acetylation, %; **deacetylation%; ***analyzed as purchased

****MWT: molecular weight

It is clear from Figure 1 that, a very wide absorption peak between 3362–3286 cm^− 1^ that signify stretching vibration of the hydroxyl group (-OH), an amine group (-NH_2_), and hydrogen bonding in the extracted chitosan (CHI-CHIV). Those are very comparable to the spectrum peak of commercial one, i.e., 3361–3283 cm^− 1^ (CHC). It is worth to notify that, not any of the presented spectra in Figure 1, even the commercial chitosan, showed any sharp peak close to 3500 cm^− 1^, which corroborates that the hydroxyl groups in positions C2 and C6 of the chitosan are engaged in intra- and intermolecular hydrogen bonds [14].

The band assigned close to 2879 cm^− 1^ can be attributed to -C-H stretching vibration, even when there is a shift of this peak according to the acid utilized in the extraction of the chitosan. The deproteinization step of chitosan can be prove by the decline in the -CH signal absorbance intensity and non-significant shift in its alignments which represent stretching vibration and the angular deformation of the -CH_2_ groups in the protein residues moieties [11, 15]. The calculated intensities values for chitosan’s -CH_2_ groups absorbance subjected to increasing deacetylation periods were 0.09034%; 0.07202%; 0.06696% and 0.03678% for the products deacetylated for 22 h; 30 h; 36 h and 40 h, respectively. The bands assigned near 1585 cm^− 1^ for the extracted chitosan and at 1591 cm^− 1^ for the commercial one can be referred to the amide-II groups and describe the -N-H bending modes.

Through the N-deacetylation of chitin, the band assigned close to 1655 cm^− 1^ is gradually diminish, while that at the range of 1584–1591 cm^− 1^ increased, demonstrating the occurrence of -NH_2_ groups [14].

The methyl group in -NHCOCH_3_ (amide bond) is estimated to be located close to 1375 cm^− 1^ in the extracted and commercial chitosan. On the other hand, oxygen stretching vibration in the glycosidic bond and the antisymmetric stretching of the C–O–C bridge are assumed to be assigned near 1150 cm^− 1^ [16, 17].

Moreover, it can be claimed that the bands related to the inorganic carbonates are similar to that of shrimp shells, assigned at 1421 cm^− 1^ and 892 cm^− 1^ with the probability of shift of those peaks based on the acid utilized in the extraction of the chitosan, as previously stated [18]. Those two vibration peaks suggest the existence of a carbonate group (CO_3_^2−^), which can be partially due to the occurrence of carbon dioxide gas that sorbed throughout the extraction procedure [19].

Additionally, the peaks at 892 cm^− 1^ can be attributed to pyranose ring stretching vibration [18].

Based on data in Figure (1-CHC) the most peaks of interest for the commercial chitosan were: 3356 cm^− 1^; 3291 cm^− 1^; 2855 cm^− 1^; 1649 cm^− 1^; 1591 cm^− 1^; 1421 cm^− 1^; 1373 cm^− 1^; 1150 cm^− 1^; 1061 cm^− 1^; 990 cm^− 1^; 892 cm^− 1^ and 438 cm^− 1^. In brief, the figures for the peaks assignment for deacetylated chitosan, under consideration, are in great agreement with the peaks of the commercial chitosan and that published in the literature [11, 20–22].

To summarize the most peaks of interest and characterizing the extracted chitosan, in general, are near the range 3360 − 3290; 2855–2879; 1584–1592; 1320–1449 besides 1420; 1348; 1150; 1078; 1035; and 417 cm^− 1^. Close figures for peaks assignments were published by Singh et al. [19].

### Degree of deacetylation (DD) [2]

As stated before, the degree of deacetylation is one of the main crucial factors that influence the quality of chitosan. The greater is the deacetylation degree, the greater is the purity of the chitosan. The degree of deacetylation is usually described as a vital parameter for verifying the thermal stability, the biological efficiency, biomedical applications, polymeric as well as the physical and chemical properties of chitosan [23–27]. Additionally, the DD is an important factor demonstrating the deacetylation process efficiency for chitin precursor. The whole experimental conditions are the principal determinate factors affecting not only the main characters of the produced chitosan but also the chitin’s deacetylation outputs. It was published that the concentration of sodium hydroxide [NaOH] is the main factor in the deacetylation stage, where the deacetylation degree escalates by rising NaOH concentration. Moreover, the escalation in reaction periods results in a rise in the DD%, Table 1. On the other hand, the rising of these two factors i.e. NaOH concentration and the deacetylation time, leads to a reduction in the intrinsic viscosity and molecular weight of the yielded chitosan [28]. Table 1 presents the molecular weight of the obtained chitosans which is smaller than 41,300 for commercial ones comparable to 15,280, 14,957, 13,349, and 14,421 for chitosans after chitin deacetylation for 22 h, 30 h, 36 h, and 40 h, respectively.

### Evaluation the degree of deacetylation (DD)

Determination of the deacetylation degree for the different variables of the extracted chitosan, in this study, is based on the ratio of the intensities of the absorption bands near 1320 cm^− 1^ and that near 1420 cm^− 1^ for each product. In the extracted chitosan spectra, the peak near 1420 cm^− 1^ for stretching vibration of -CH_2_ groups in -CH_2_ OH at C-6 is assumed to be one of two standard peaks, while the peak close to1320 cm^− 1^ is the second and corresponding to the characteristic band of amide groups (-OH, -NH_2_, -CO) [20].

First, the degree of acetylation magnitudes of chitin, (DA %), can be computed, as presented in Eq. (2): [17, 20]and [29].

2 $$D A \%=31.92 *(A 1320 / A 1420)-12.20$$

3 $$D D \%=100-D A \%$$

Secondly, the degree of deacetylation percentage, (DD %), is calculated according to the Eq. (3) [30].

The acetylation DA% and deacetylation DD% data reached for the different acquired chitosan besides that for the commercial one are presented in Table 1.

It is assumed that the ratio relets the intensities of two bands near 1320 / 1420 presents a minor experimental error for evaluating the chitin deacetylation, despite of the method and the nature of the matter [2, 31].

The degrees of deacetylation, DD%, escalate as the decline in the number of the acetyl groups and attribute to the protonation of the -NH_2_ groups on the carbon 2 (C2) position of the replicating D-glucosamine units. This proposing distinguished quality of chitosan product [20].

According to Joseph et al. and based on the data obtained for deacetylation, the extracted chitosan under consideration can be classified as a product with high deacetylation degree (HDD) (70–99%) [32].

It is reported that the deacetylation degree of the commercial chitosan ranges, mainly, from 74 to 80% [33]. It is worth to state that the purchased one and used for comparison in this study has DD % near 76%. Compared to the figures obtained in the present study, this value is comparable to the extracted chitosan which subjected to increasing deacetylation periods (Table 1).

### Scanning electron microscopy (SEM)

**Fig. 2:**
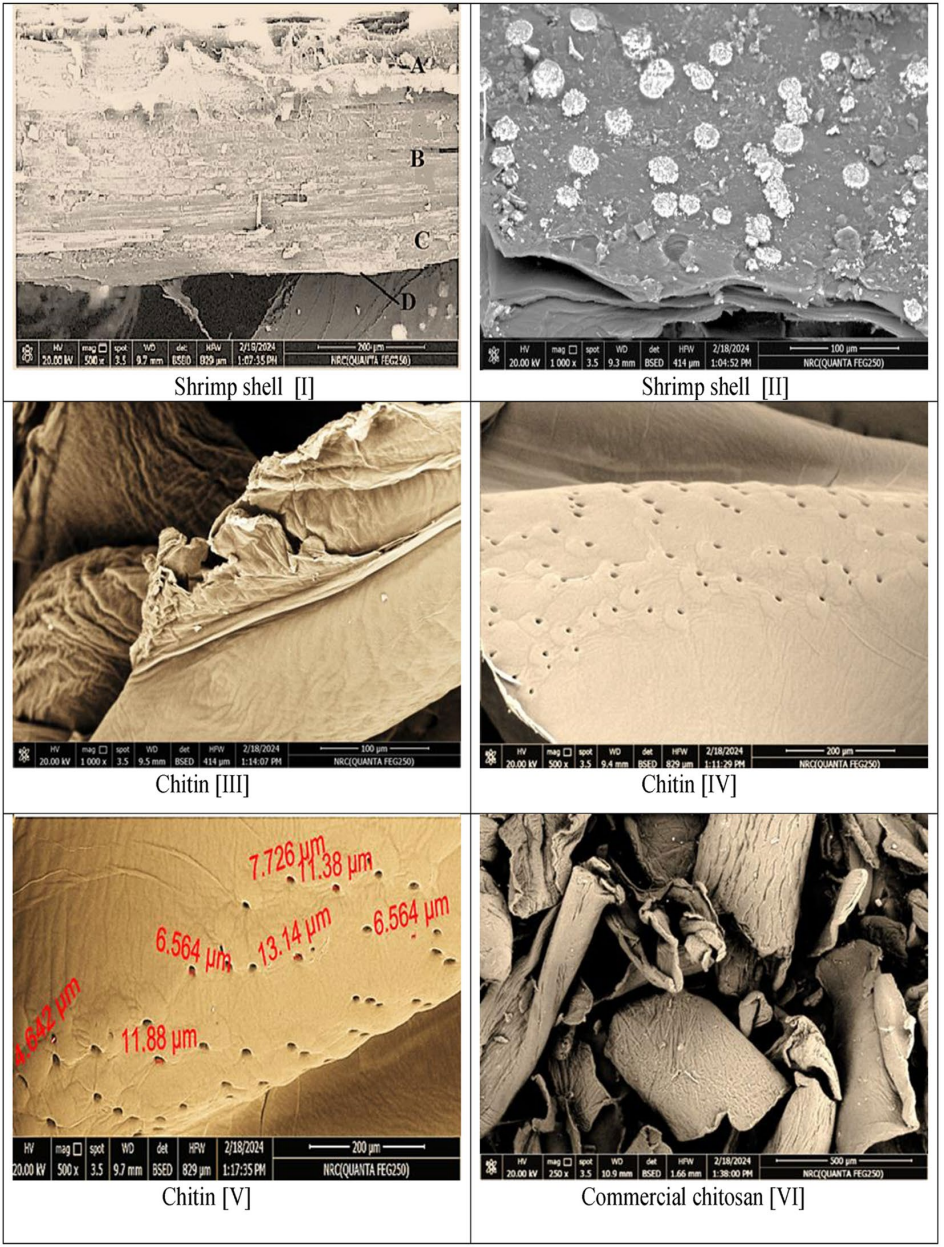

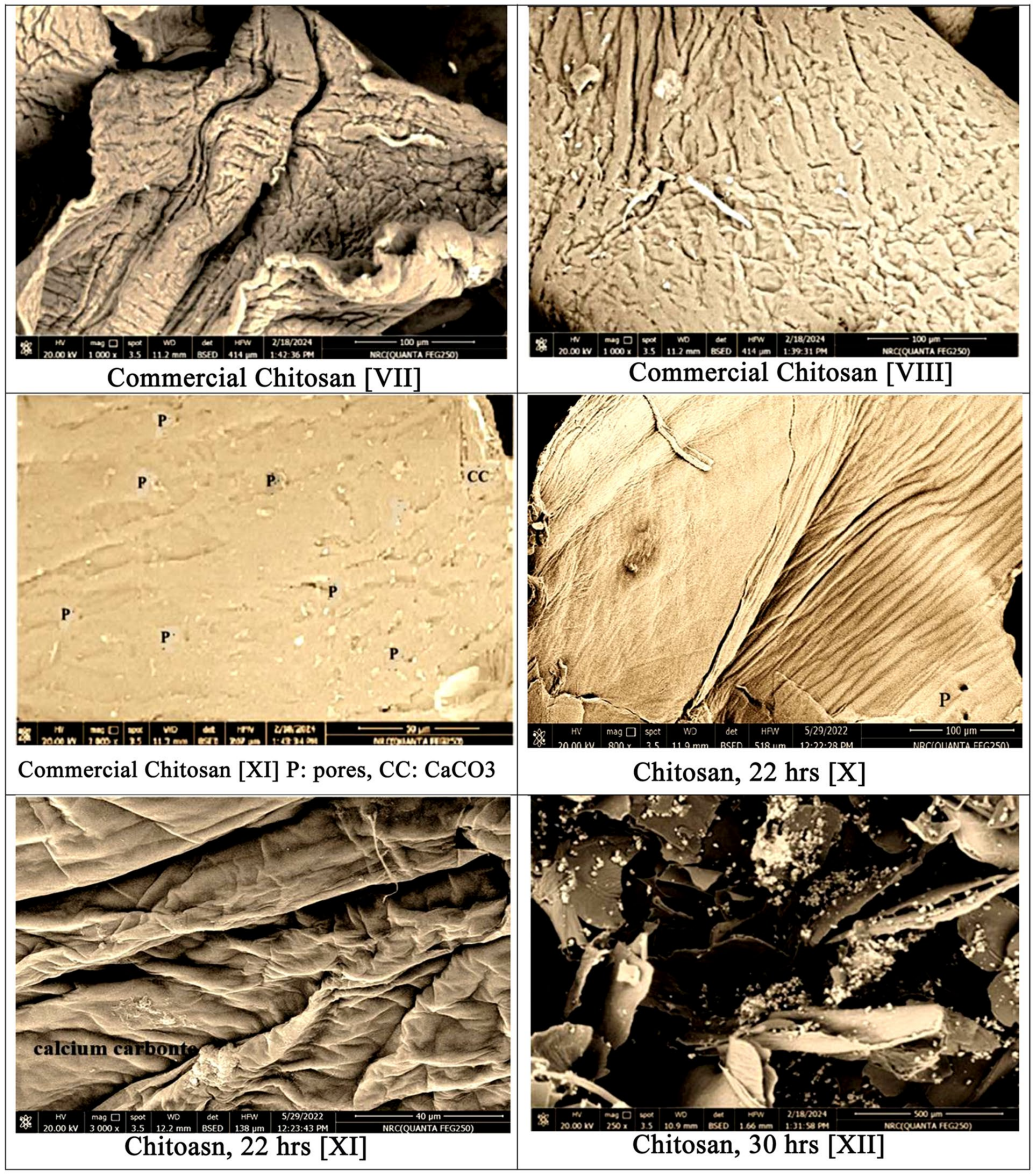

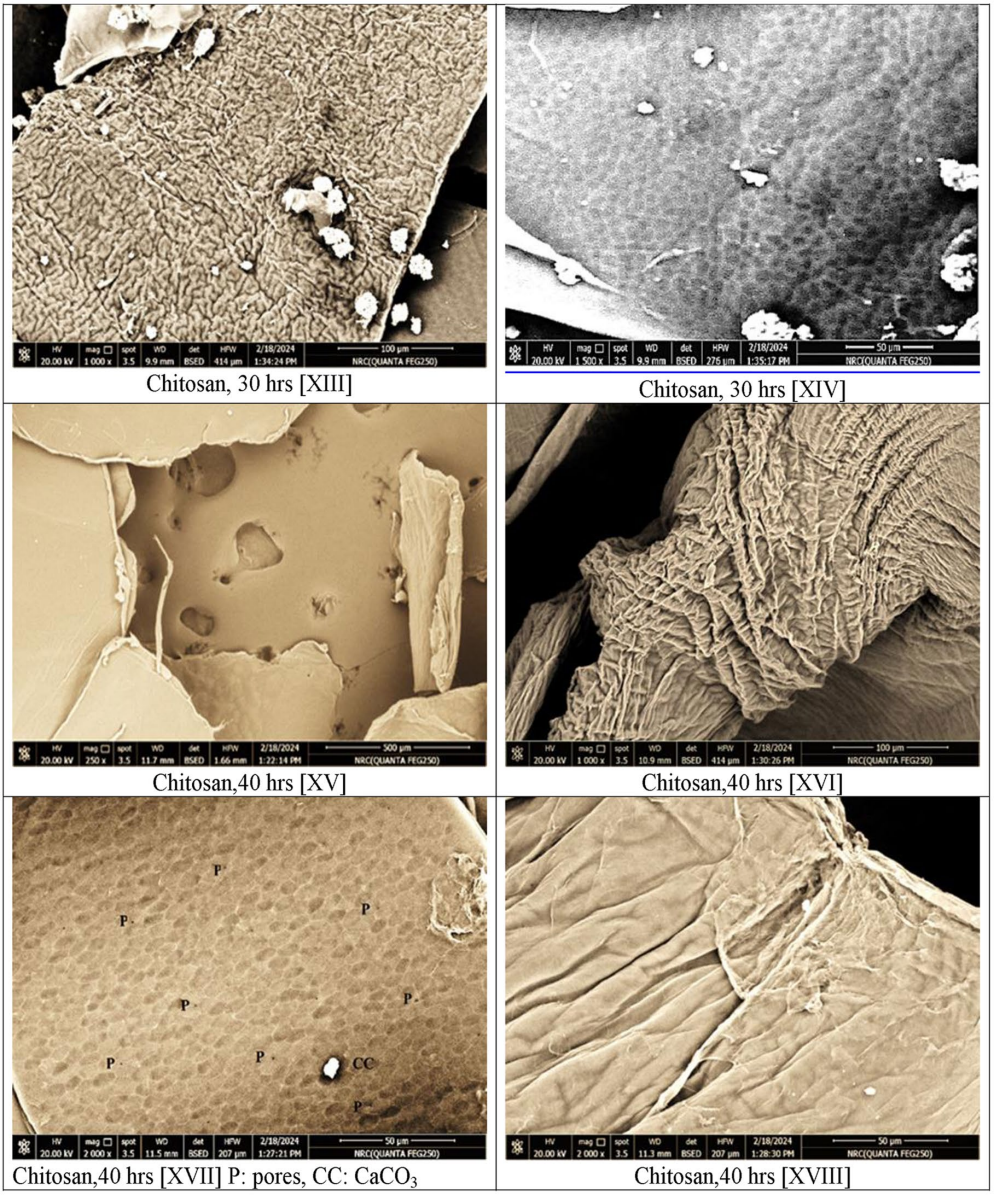
Scanning Electron Microscope examinations with magnification 250x, 500x, 800x, 1000x, 1500x, 2000x and 3000x for shrimp shell [I & II], chitin [III-V], commercial chitosan [VI-IX], extracted chitosan deacetylated for 22 h [X-XI], extracted chitosan deacetylated for 30 h [XII-XIV] and extracted chitosan deacetylated for 40 h [XV-XVIII]

The microstructure of the shrimp shells, under consideration, as precursor for chitin and chitosan as well as the impact of each processing step on the morphology of every product were examined by SEM.

(Figure 2-I) presents the topography of shrimp shell waste, under consideration, which indicates main four layers for the shell, namely, the epicuticle [A], the exocuticle [B], the endocuticle [C], and inner membrane [D]. The three former layers are observable as definite layers in the micrograph while the inner membrane is very thin, discontinuous, and hard to detect. The shell architecture looks to be fibrous. The thickness of the three layers is arranged as exocuticle > epicuticle > endocuticle. The same arrangement was reported by Xu et al. [34].

Shrimp shells show a heterogeneous morphology distinguished by a compacted assembly with well-distinct chips-like shapes that have white spots. Additionally, the shell seems to possess a tough overlay with very rare pores which is attributed to the existence of protein and mineral components that appeared as white spots. Figures (2-II) [35, 36].

The internal structure of chitin and chitosan was examined, also, by scanning electron microscope. Figures (2, III-XVIII) presented the SEM photos of chitin, commercial and extracted chitosan with several magnifications and at various zones.

The obtained chitin past demineralization and deproteinization processing of the shrimp shells possessed irregular smooth microstructures. Moreover, the micrograph presents blocky fiber arrays besides the uneven distinct horizontal fiber structures. Figures (2-III). However, the topography of chitin and /or chitosan is dependent upon the type and the method of processing of the shells [37–40].

Chitin is displaying, also, a compactly flattened morphology, containing round pores collection with thick walls surrounding the pores and the remains of the spouts within (Fig. 2-IV). The white cake-like locations in the shells are assumed to be substituted by the rounded pores as a result of the deletion of protein and calcium carbonates [CaCO_3_] crystals after the demineralization and deproteinization steps [39]. Comparable remark was stated by Yen et al. (2009) [41], Arbia et al. [42], and Muzzarelli et al. [43]. In this examined area of chitin, the pores sizes are ranged from 4.642 μm to 13.14 μm (Fig. 2-V).

An outline SEM micrograph for the commercial chitosan is presented in Figure (2- VI) and pairs of detailed photos are depicted in (Figure 2 VII-IX). The former image, with magnification 250x, demonstrates discontinuous, uneven blocky fiber structures, crumbly flakes, and even soft areas. At 1000x magnification for a selected slice of commercial chitosan, fibril arrangements can be differentiated, in addition to rough, disordered, disorganized, some cracks, and hardly detected very small pores with distorted topography (Figure 2-VII). Moving to another area: lump and little white gatherings similar to that manifested in shrimp shell microstructure were detected in (Figure 2-VIII). Also, at 1000x magnification, at an additional area of the commercial chitosan, a more or less even flattened surface with micro-pores can be manifested. Figures (2, IX). Similar results were reported [16, 44].

The architectures of the extracted chitosan were examined, also, by SEM and the micrographs with different magnifications, different areas, and those subjected to escalating deacetylation periods are presented in Fig. 2 [X–XVIII].

The microstructures at different parts for chitosan generated from the deacetylation of chitin for 22 h are presented in Fig. 2 (X, XI) with magnifications 800x & 2500x, respectively. At 800x magnification more or less even flattened surface besides regular rough smooth microstructure with very rare micro-pores can be manifested (Figure 2-X). On the other hand, at 3000x and at other spots: folded surface, irregular pattern, with some longitudinal spaces in-between, little threads, and no obvious pores can be distinguished (Figure 2-XI).

For chitosan deacetylated for 30 h, the examination of the outcome at magnification 250x display asymmetric arrangements with irregular flacks. The surfaces are of various particle sizes and various lumpy, rough fissures, and irregular pattern, with some void spaces, little tiny threads, and no distinct pores can be differentiated. Figures (2-XII).

As the deacetylation degree after 30 h recorded only ≈ 78.23%, Table 1, the micrograph, at 1000x magnification in (Figure 2-XIII) for the extract chitosan showed traces of non-deacetylated chitin. Examining other area, at 1500x magnification, showed uniformity brick like shape topography with trace of carbonate residues and micropores which can be hardly viewed [16, 45]. (Fig. 2- XIV).

**Table 2:** Atomic and mass percentages of shrimp shell, extracted chitin, commercial and deacetylated chitosan based on EDX analysis

Element	Mass, %				Atomic, %			
	SS	CT	CHC	CH	SS	CT	CHC	CH
C	27.28	44.9	37.65	33.9	36.54	55.19	45.09	41.22
O	56.33	45.55	60.32	63.29	56.65	42.04	54.23	57.77
Na	0.58	0.06	N.D.	0.01	0.4	0.04	N.D.	0
Mg	1.01	0.39	0.27	0.46	0.67	0.24	0.16	0.28
P	1.84	0.78	0.18	0.66	0.95	0.37	0.08	0.31
K	0.18	0.56	0.17	0.07	0.07	0.21	0.06	0.03
Ca	9.68	0.4	0.27	0.14	3.89	0.15	0.10	0.05
Mn	0.38	0.83	0.23	0.1	0.11	0.22	0.06	0.03
Fe	0.28	0.66	0.20	0.09	0.08	0.17	0.05	0.02
Ni	0.54	1.08	0.30	0.17	0.15	0.27	0.07	0.04
Cu	1.28	2.57	0.21	0.56	0.32	0.6	0.05	0.13
Zn	0.63	2.24	0.21	0.56	0.73	0.51	0.05	0.12

SS: shrimp shell – CT: chitin- CHC: commercial chitosan -

CH: extracted chitosan after deacetylation for 30 h- ND: not detected

The microstructures at different parts of chitosan generated from the deacetylation of chitin for 40 h are presented in Fig. 2 (XV-XVIII) with magnifications 250x, 1000x & 2000x, respectively. An overview at magnification 250x, for the extracted chitosan and deacetylated for 40 h, the sample shows predominant smooth and plane region. A smooth polymer with chips-like plates and distinguished pores can be viewed also within this area (Fig. 2-XV).

Examination at another spot and at magnification 1000x indicates folded zone with unevenness structure, tough, irregular arrays, clear fissures and no definite pores can be distinguished. The micrograph discloses, also, a rough, disordered, disorganized and crumbled microstructure surface (Figure 2-XVI).

Applying a higher magnification 2000x, reveals a soft and homogenous separated area demonstrating a plywood pattern structure. Traces of the white lumps as that of the shrimp shell can also be detected, in this area of examination. Moreover, pairs of micro-pores can be detected in this area (Fig. 2-XVII). The lack of homogeneity in the chitosan surface with a flattens area and roughness of another can be attributed to incomplete extraction of the chitosan from shrimp shells.

The micrograph of (Fig. 2-XVIII) at 2000x magnification in another part presents, also, that chitosan has an uneven and wavy identities which were affected by the deacetylation treatment of chitin. This can confirm the impact of deacetylation time on the properties and morphology of the chitosan output [46].

Based on the SEM examination the following remarks can be summarized:

- Generally, it can be confirmed that the extraction technique of chitosan displays variations in the shape and morphology of the final product.
- There is no entire elimination for all minerals, protein, and impurities from the precursor shrimp shells.
- The lack of homogeneity in chitosan’s surface with smooth segment and rough area is the indications of part split-up and signify the incomplete extraction process.
- The impact of the used chemical concentration and processing period applied influence the architectures microstructure of the final product.

**Fig. 3:**
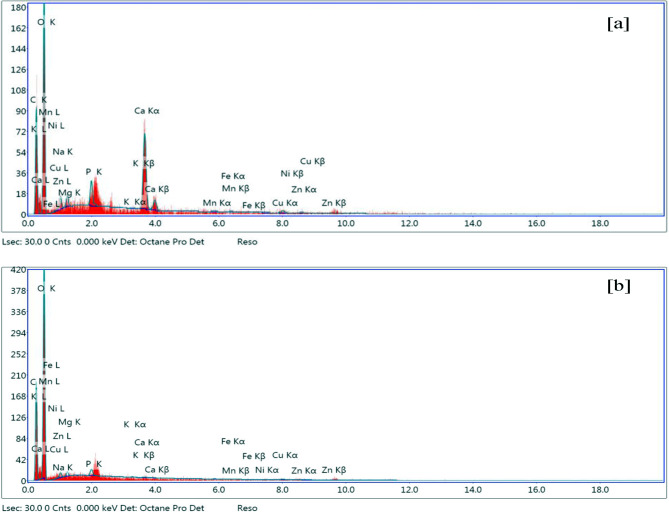

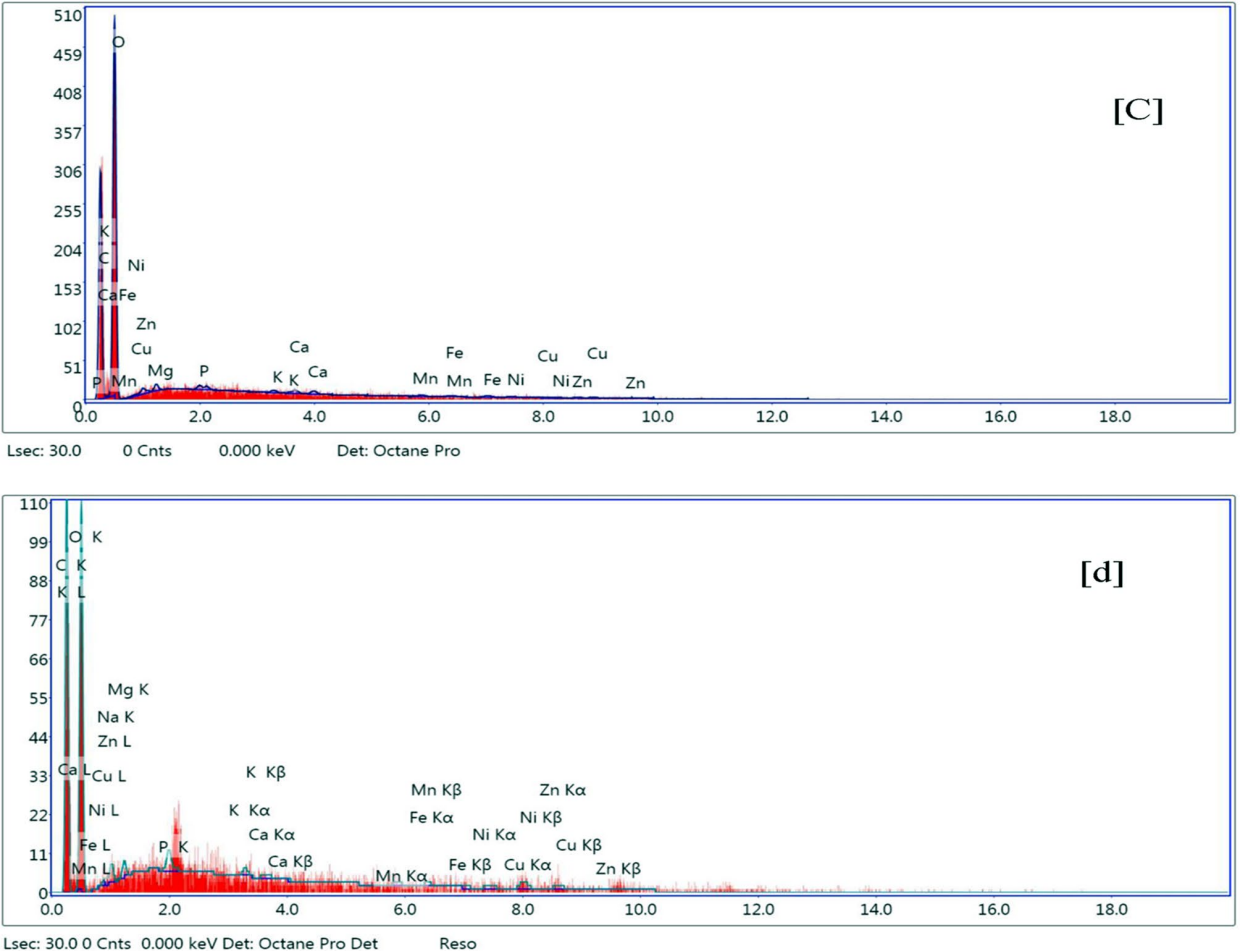
EDX spectra analyses for (**a**): shrimp shell, (**b**): chitin. EDX spectra analyses for (**c**): commercial chitosan (**d**): extracted chitosan after chitin deacetylation for 30 h

The spectra of Energy dispersive analyses of X-ray spectroscopy (EDX) for shrimp shell, extracted chitin, commercial chitosan, and deacetylated one for 30 h were presented in (Figure 3 a-d and Table 2). The elemental constitution of the four stuffs corroborated that the principal peaks in the four spectra are for carbon (C) and oxygen (O). The intensity of the carbon peaks was maximum for chitin, (Fig. 3b), followed by commercial and extracted chitosan, (c & d), while, the lowest peak intensity was for the shrimp shell (Fig. 3a). This can be attributed to the concentration of carbon contents after demineralization and deproteinization followed by dissolution due to deacetylation processes. It is clear from (Figure 3 a-d) and Table 2 that the mass of calcium (Ca) % is found in the shrimp shell near 10% and at concentrations less than 1% in both chitin and two chitosans. Concerning the presence of traces of Ca in chitin and chitosan outputs, (Fig. 3, b, c &d), Li et al., [40] and Guan et al., [47], proposed that, the pureness of the extracted chitin and chitosan is based on its origin, demineralization and deproteinization applied techniques, chemicals used [20] and processing period [40].

**Table 3:** UV–Vis absorbance peak’s assignments and character for extracted chitosan acquired due to deacetylation of chitin for 22 h, 30 h, 36 h, 40 h and commercial chitosan

Stuff	Absorbance, nm	Peak’s character	
CH22	238	strong	sharp
	289	shoulder	
CH30	245	medium	sharp
	290	shoulder	
CH36	242	medium	sharp
	290	shoulder	
CH40	242	medium	sharp
	292	medium	broad
	346	medium	broad
CHC	248	weak	broad

**Fig. 4:**
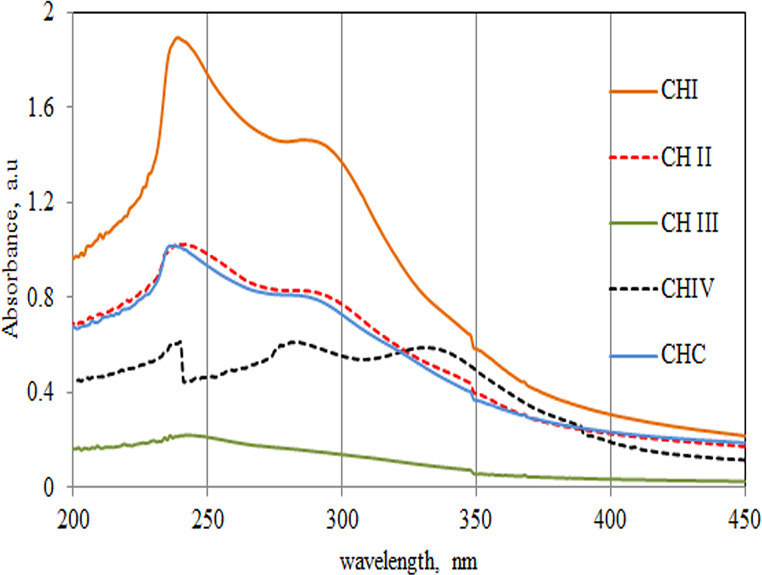
UV–Vis spectra for the deacetylated chitosan for 22 h (CHI); 30 h (CHII); 36 h (CHIII), (CHIV) and for commercial chitosan (CHC)

**Fig. 5:**
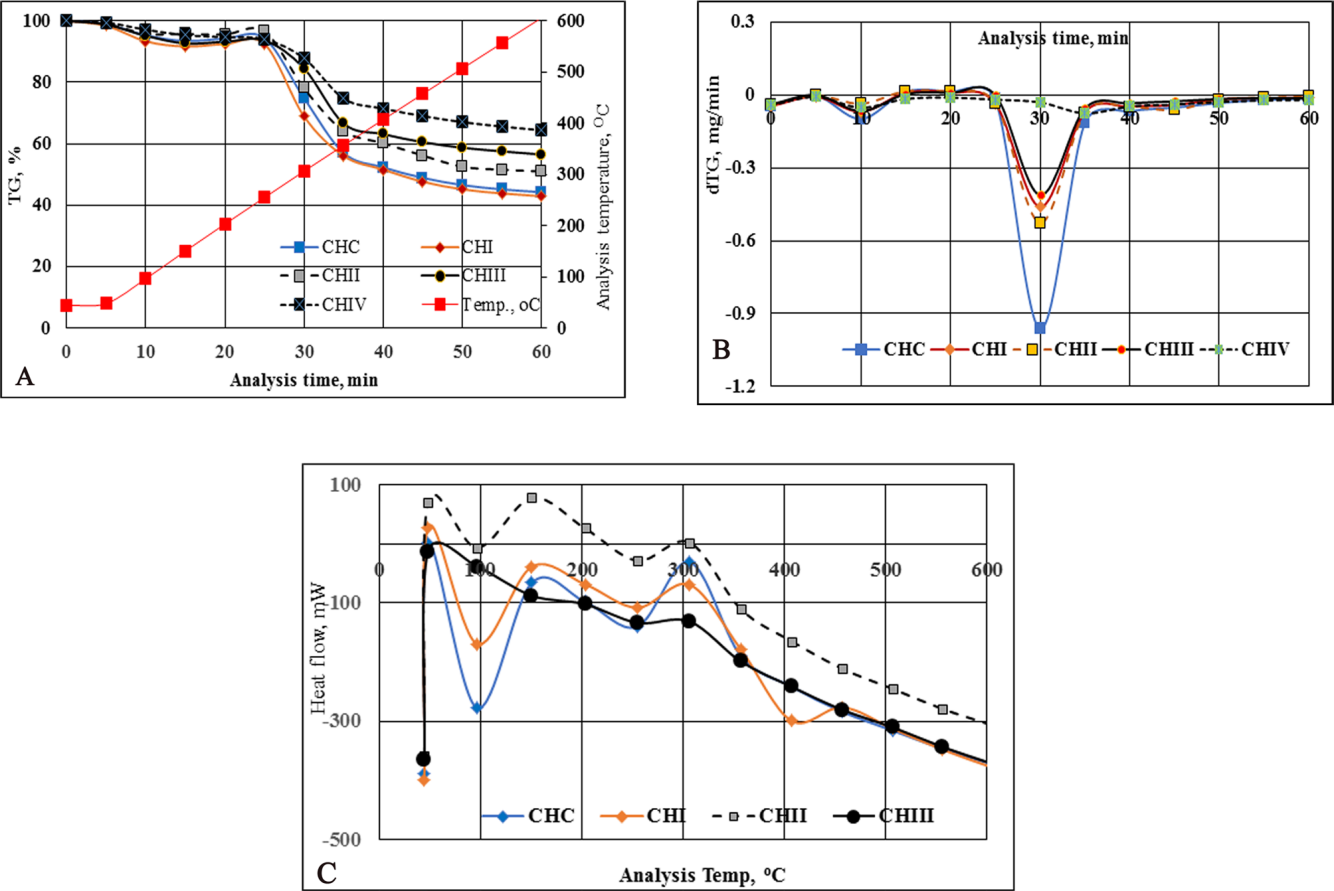
TG [**A**], DTG [**B**] & DSC [**C**] thermograms of commercial chitosan and extracted ones generated from chitin deacetylation for escalated periods

This result proves the incapacity of diluted acid to remove all minerals from the shrimp shells, contrary to concentrated acids can leave just a few minerals traces.

It is clear from Table 2, also, that there are insignificant variations in the composition of the commercial chitosan compared to the extracted chitosan under consideration. It worth to state that these very minute differences can be referred to shrimp origin, demineralization and deproteinization applied techniques, chemicals used processing period and even the shelf shipping conditions and period [40, 47].

According to the United States Environmental Protection Agency (U.S. EPA), and the International Agency for Research on Cancer (IARC): arsenic, cadmium, chromium, lead, and mercury, due to their high degree of toxicity, these five elements classified among the main concern metals that are of great public health significance. They are all systemic toxicants that are known to provoke multiple organ damage, even at lower levels of exposure [48]. Consequently, and based on EDX analysis, this confirms that the extracted chitosan under consideration, in connotation, would be a safe and non-toxic product when used in medical, pharmaceutical, biological, and food stuff industries.

### Ultraviolet-visible spectroscopic analyses

The Ultraviolet-Visible (UV–Vis) spectra for extracted chitosan acquired from chitin deacetylated for 22 h, 30 h, 36 h, 40 h, and commercial chitosan, are presented in (Figure 4). The UV–Vis spectra for each stuff display distinguishing absorption bands. Table 3 depicts the absorption maximum for the extracted chitosan generated from deacetylation for 22 h (CHI); 30 h (CHII); 36 h (CHIII), 40 h (CHIV), and commercial chitosan (CHC).

The absorption spectra of UV–Vis for the five stuffs are comparable and allocated peaks λ at 238, 245, 248, 242, and 242 nm for extracted chitosan acquired from deacetylation for 22 h, 30 h, for 36 h and 40 h and for CHC respectively. Even they differ in their intensities, it should be stated that the extracted chitosan generated after chitin deacetylation for 40 h has a third peak at λ 346 nm. It was reported that the peak between 300 and 370 nm is characteristic for chitosan [49]. Little bite shift can happen to the low limit at λ 290 nm. On the same trend, the peak at λ 300–360 nm contributes to the absorption linked to the direct electronic π-d orbitals and is entitled as the Soret band [50].

The sharp peaks for chitosan in the UV -Vis can verify their promise usages in waste water handling, nanoparticle invention and others [51].

#### Thermal characterizations

The Thermogravimetric analysis (TGA) curves, the mass changes TG in % versus sample temperature, °C, are applied to present the changes in material composition and its thermal stability. On parallel, DTG curves apply to ascertain the number of thermal consequences to which the material has been exposed, since each DTG peak, at a given temperature provides the rate of mass loss (mg/min) through its temperature range, (*Ton*,* Tmax.*,* Toff*), where *Ton*, is the temperature of onset mass loss; *Toff* is the temperature of offset mass loss and *Tmax* is the temperature of the maximum mass loss rate within that peak. Thermogravimetric analyses (TG & DTG) were achieved systematically for commercial chitosan (CHC), for extracted chitosan intended from increasing deacetylation periods for: 22 h (CHI); 30 h (CHII); 36 h (CHIII), and 40 h (CHIV) under the same analyses conditions. The thermal analyses for the commercial chitosan were performed for the sake of comparison. The data acquired are presented in (Fig. 5) and Table 4.

**Table 4:** Thermal analyses data for commercial and the acquired extracted chitosan as a function of increasing the deacetylation periods

Deacetylation period, (hours)	Peak array	Temperature, of degradation peaks, (°C)			Mass loss at maximum, (%)	Total mass loss, (%)
		On set	Maximum	Off set		
Commercial*	first	48.10	87.24	131.80	7.09	52.50
	second	278.83	305.26	323.87	33.17	
22	first	44.57	82.54	121.03	8.73	52.00
	second	268.03	300.20	32.07	32.07	
30	first	44.86	80.92	105.10	5.88	37.50
	second	265.77	303.40	325.92	29.86	
	third	436.29	476.32	496.74	22.00	
36	first	43.52	89.30	132.32	8.61	41.00
	second	285.83	313.34	330.56	23.12	
40	first	40.85	79.67	147.86	5.61	36.32
	second	282.94	310.65	329.89	15.17	

*commercial chitosan applied without any treatment

It is claim that the chitosan polymer, in general, displayed four significant mass loss steps attributed to: water moisture loss, degradation of the organic moiety, including the protein remained in chitosan past the all-extraction steps, the decarboxylation of calcium carbonate, degradation of the inorganic matters and/or recrystallization of any amorphous inorganic substances. Similar trend was reported for chitin [6, 52].

The TG curve for commercial chitosan demonstrates two steps of the polymer degradation. The first step began from 44.66 °C up to 158.91 °C with mass loss 6.08%. The corresponding DTG peak exhibits onset at 48.10 °C, the temperature of the maximum mass loss rate within that peak at 87.24 °C, and offset at 131.89 °C with mass loss at a maximum of 7.09%. This mass loss is attributed to the water physically adsorbed inner and at the surface of CHC. The second step with a high mass loss of 49.97% started at 146.13 °C and ended at 605.61 °C. The parallel DTG curve displays onset at 278.83 °C and offset at 323.87 °C with swift mass loss of 33.17% at *Tmax* 305.26 °C signifying the decomposition of the polysaccharide and protein moieties [6] Figure (5), Table 4.

The chitosan CHI, CHIII, and CHIV are disclosed in the same manner as commercial ones, and undergo the two steps of mass loss. On the other hand, the chitosan CHII showed three steps of mass loss. The additional third step exhibits onset at 436.29 °C and offset at 496.74 °C with a mass loss of 2.196% at *Tmax* 476.32 °C. This step can be attributed to the thermal dehydroxylation of calcium hydroxide [Ca (OH)_2_] formed by increasing the deacetylation time, and took place in the region between 400 °C and 600 °C, followed by the decarboxylation of the formed carbonated phases and calcite up to 600 °C according to Eq. (4) & Eq. (5), respectively [53, 54].

4 $$\mathrm{Ca}(\mathrm{OH})_2 \longrightarrow \mathrm{CaO}+\mathrm{H}_2 \mathrm{O}$$

$${\text{Calcium hydroxide}} \quad\quad\quad{\text{Calcium oxide}} + {\text{Water}}$$

5 $$\mathrm{Ca}(\mathrm{CO})_3 \longrightarrow \mathrm{CaO}+\mathrm{CO}_2$$

$${\text{Calcium carbonate}} \quad\quad\quad{\text{Calcium oxide}} + {\text{carbon dioxide}}$$

Based on thermal analyses data, it can be deduced that a process of chitosan extraction has an impact on their thermal stability. It is clear from Table 4 that the chitosan CHIV is most thermally stable compare to the other ones and even to the commercial chitosan. The chitosan II loses its mass gradually, past three degradations showing lowest mass loss i.e. 23.12%. Similar trends were previously published [55–57].

Parallel to the thermal analysis (DTA&DTG), the differential scanning calorimetry (DSC) curves for the different extracted chitosan were performed under Nitrogen atmosphere. (Figures 5). Two decomposition stages for the all-chitosan preparations were disclosed which can be briefed as follows: the first one at ~ 80 °C, compared to the commercial CHC at ~ 86 °C and can be attributed to an endothermic reaction comprises the release of moisture surface water and hydrogen-bonded water. The second is a strongly exothermic peak that disclosed between 304 and 313 °C compared to 309 °C for chitosan CHC. This degradation second step is attributed to the degradation of the rest cross-linked chitosan skeleton [58]. Hence, it can be noted that the thermal decomposition of the chitosan polymers, under consideration, happened in two marked steps.

The thermal destruction progression of chitosan leans to be quiet beyond 356.74 °C. Though, this was a minor descending inclination near 600 °C, and can be attributed to the residual carbon content [59], and comprises 13.01%, 13.22%, 10.07% and 9.38% for CHI, CHII, HIII and CHIV, respectively and comparable to commercial one (CHC), (Figure 5).

Hence, it can be noted that the thermal decomposition of the chitosan polymers, under consideration, happened in two marked steps. Based on the data acquired for the TGA, TGD, and DSC analyses it can be claimed that the extracted chitosan from shrimp shell is characterized by an acceptable thermal stability and can, practically, represent a valuable and cost-effective natural biosorbent for some hazardous and radioactive waste streams, in addition, to the other numerous agriculture, industrial and medical applications.

According to the data obtained, based on thermal analyses, the following conclusions can be summarized:

- The method of chitosan extraction has an impact on their thermal stability.
-The water moisture loss happened in between ~ 44 °C up to ~ 158 °C comparable to that of commercial one Table 4.
- The mass loss percentages due to water moisture depletion are in range from 5.61 − 8.77% with mean value 7.31% which is not far from the commercial chitosan (7.09%).
- The mass losses next to the first step, are largely, assigned to a complicated processes involving: dehydration of the polysaccharide rings, depolymerization and decomposition of the deacetylated moieties of chitosan, besides, to dehydroxylation and destruction processes.
- As of the extensive magnitude of inter- and intramolecular hydrogen bonding in chitosan; accordingly, it needs additional energy to fracture [60].
-The acceptable thermal stability of the developed chitosan, under consideration recommend it as effective biosorbent for treatment of some hazardous and radioactive waste streams, even, at temperature near 100 °C.
-The final mass loss for the chitosan is above 600^°^C which assumed to be due to the decomposition of the traces of CaCO_3_ decomposed to CaO and CO_2_ [61].
-The adequate thermal stability of chitosan under consideration can be attributed to the intramolecular and intermolecular hydrogen bonds [62].

## Conclusion

Based on the experimental data reached, the following conclusions can be reported: The conventional chemical method applied is a reliable, straightforward, and cost-effective technique for extracting chitosan from shrimp shell waste due to the cheap chemical used, non-sophisticate, and consistent processes. Practically, increasing the deacetylation time of chitin significantly influences the Degree of Deacetylation (DD) of chitosan and, consequently, affects its spectroscopic characteristics. Moreover, the spectroscopic tools used are effective for indicating the impact of various deacetylation times on the configuration of the extracted chitosan. Additionally, the results reached confirm that the extracted chitosan, under consideration, is pure, ultrafine, and non-toxic, with an acceptable degree of deacetylation (DD) of nearly 80%. The extracted chitosan exhibits acceptable heat resistance, showing no thermal degradation until it reaches 300 °C. This resistance is comparable to that of commercial chitosan. The configuration of chitosan closely resembles that of cellulose, with the distinction that the hydroxyl group at the C-2 position in cellulose is replaced by an amino group in chitosan. This -NH_2_ group enables chitosan to effectively scavenge pollutants, such as heavy metals and radionuclides, from waste solutions.

As waste recycling becomes of global interest, shrimp shell wastes can be utilized more effectively by extracting chitosan, which can then be applied in a wide variety of everyday applications. From an environmental point of view, stimulating marine biomass can yield significant economic and environmental benefits for industries, including the fishery sector. Utilizing shrimp waste as a source of chitosan extraction on an industrial scale would help address the accumulation of waste generated by processing plants, presenting a challenge for both the industry and community health.

Future projects are planned to explore the combination of the extracted chitosan with certain cellulose derivatives, either as a backbone or a cross-linking agent. These combinations are expected to produce various hydrogel products with promising economic value that can be applied in many areas of daily life. It is recommended, also, that more studies can focus on investigating the antimicrobial and antioxidant properties of chitosan extracted from Egyptian shrimp shell wastes.

## Data Availability

The data will be available on your request.
